# Supplementary material for: Vitamin D Modified DSS-Induced Colitis in Mice via STING Signaling Pathway
Source: Biology (Basel). 2025 Jun 18;14(6):715. doi: 10.3390/biology14060715 (PMC12190092; doi:10.3390/biology14060715)
Supplement: Supplementary file 1 [file biology-14-00715-s001.zip › Supplementary Materials S1: 16s-report of gut microbiota/Groups/diff_analysis/UnivarTestGMM/figure/barplot/top20log.pdf]

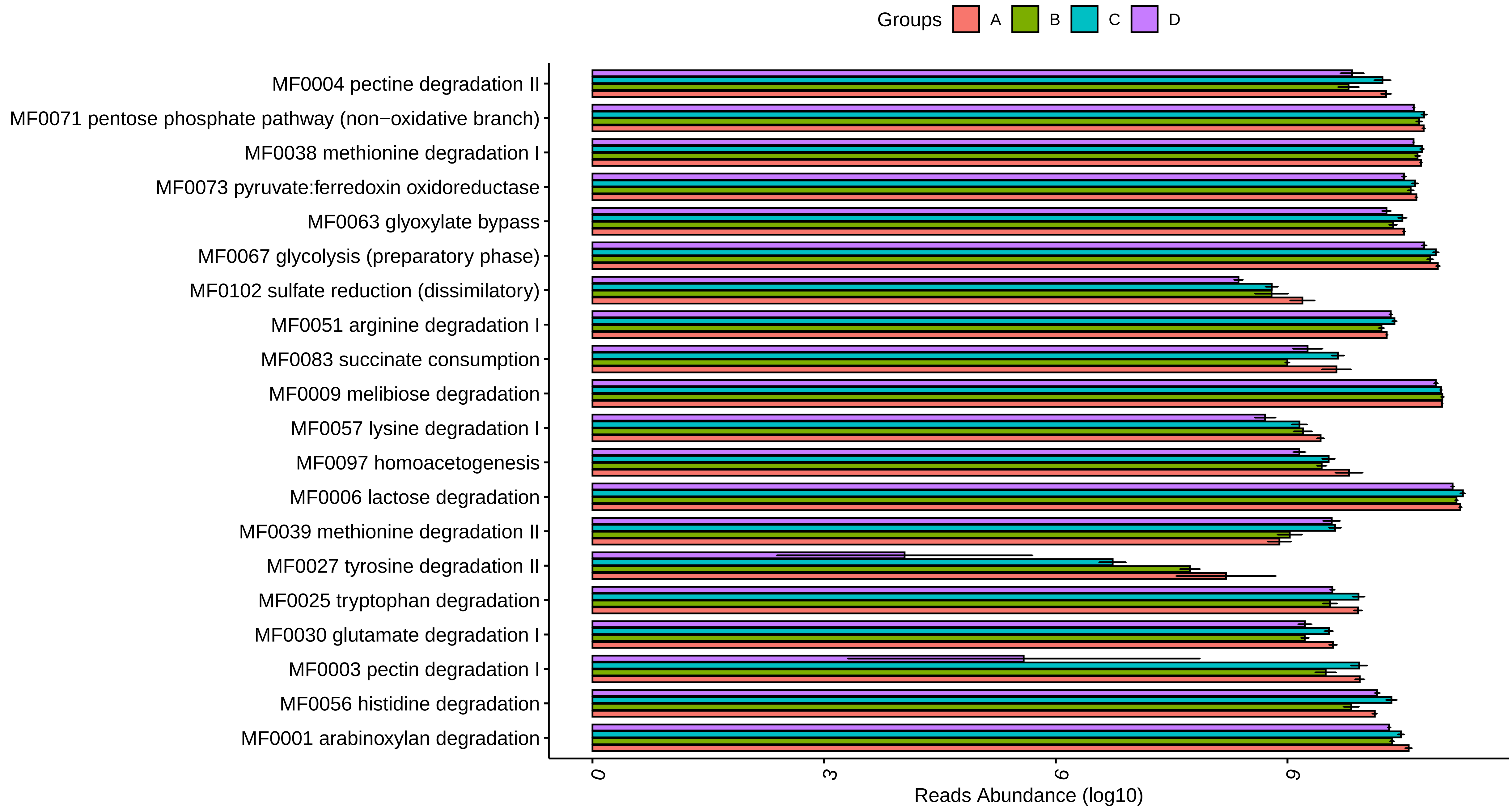
Groups
A
B
C
D
MF0004 pectine degradation II
MF0071 pentose phosphate pathway (non−oxidative branch)
MF0038 methionine degradation I
MF0073 pyruvate:ferredoxin oxidoreductase
MF0063 glyoxylate bypass
MF0067 glycolysis (preparatory phase)
MF0102 sulfate reduction (dissimilatory)
MF0051 arginine degradation I
MF0083 succinate consumption
MF0009 melibiose degradation
MF0057 lysine degradation I
MF0097 homoacetogenesis
MF0006 lactose degradation
MF0039 methionine degradation II
MF0027 tyrosine degradation II
MF0025 tryptophan degradation
MF0030 glutamate degradation I
MF0003 pectin degradation I
MF0056 histidine degradation
MF0001 arabinoxylan degradation
0
3
6
9
Reads Abundance (log10)
